# Application of a virtual reality tracker-based system to measure seated postural stability in stroke patients

**DOI:** 10.1186/s12984-022-01052-0

**Published:** 2022-07-14

**Authors:** Huey-Wen Liang, Tzu-Ling Tai, Yue-Hua Li, Ying-Chun Chen

**Affiliations:** 1 Department of Physical Medicine and Rehabilitation, National Taiwan University Hospital and College of Medicine, Taipei, Taiwan, ROC; 2 Department of Physical Medicine and Rehabilitation, National Taiwan University Hospital, Taipei, Taiwan, ROC

**Keywords:** Postural stability, Sitting, Stroke, Virtual reality

## Abstract

**Background:**

Postural stability while sitting is an important indicator of balance and an early predictor for future functional improvement in neurorehabilitation, but the evaluation is usually dependent on clinical balance function measures. Meanwhile, instrumental posturography has been used widely to obtain quantitative data and characterize balance abilities and underlying control mechanisms, but not as often for sitting balance. Moreover, traditional kinetic methods using a force platform to test sitting stability often require modification and are costly. We proposed a tracker-based posturography with a commercial virtual reality system, the VIVE Pro system (HTC, Inc. Taiwan), to record the trunk displacement (TD) path with a lumbar tracker for evaluation of sitting stability. The goals were to test the reliability and validity of the TD parameters among stroke patients.

**Methods:**

Twenty-one stroke individuals and 21 healthy adults had their postural sway measured with this system under four sitting conditions, i.e., sitting on a solid surface or a soft surface, with eyes open or closed. The test–retest reliability of the TD parameters was evaluated with intraclass correlation coefficients in 22 participants. We also tested the discriminative validity of these parameters to discriminate between stroke and healthy controls, and among four sitting conditions. Furthermore, the TD parameters were correlated with the three balance function tests: the Berg Balance Scale (BBS), the Postural Assessment Scale for Stroke Patients (PASS) and the Function in Sitting Test (FIST).

**Results:**

The results indicated that the TD parameters obtained by tracker-based posturography had mostly moderate to good reliability across the four conditions, with a few exceptions in the solid surface and eyes open tasks. The TD parameters could discriminate the postural stability between sitting on solid and soft surfaces. The stroke group had more seated postural sway than the control group, especially while sitting on a soft surface. In addition, velocity measures in the sagittal and frontal planes had moderate to high correlations with the PASS and BBS scores.

**Conclusions:**

This tracker-based system is a cost-effective option for the clinical assessment of body stability for stroke patients in a seated position and shows acceptable reliability and validity.

## Background

Achieving good sitting balance is one of the important milestones for stroke patients receiving neurological rehabilitation. More than 90% of patients can achieve unsupported sitting for one minute, but there is a great variation in the time required, depending on the disease nature and the stroke size and location [1]. Moreover, individuals who develop good sitting balance early during rehabilitation have better functional assessment outcomes than those with poor sitting balance [2–4]. Even for those who have limited functional recovery and cannot become ambulatory, the ability to maintain good balance while sitting is essential to perform basic self-care activities independently, such as eating, dressing and toileting. Therefore, it is crucial to quantify the sitting balance for stroke patients during the rehabilitation process.

The most commonly used clinical assessments of sitting balance for stroke patients include stand-alone and sitting-specific measures or global balance function measures that include sitting as parts of the balance or motor evaluation [5]. These measures can be integrated into clinical assessment, but are less well-validated than the measures for standing balance, even the stand-alone sitting scales. One systematic review identified 14 sitting balance measures, only five of which were stand-alone sitting balance scales [5]. This review assesses their methodological quality and concludes that no single scale has sufficient psychometric properties to enable recommendation as a preferred tool for measuring sitting balance among stroke survivors. Another option is to use instrumental posturography with a range of quantifiable measures, such as kinetic measures, kinematic measures, or measures of muscle activity [6]. Posturography can characterize the balance abilities and underlying control mechanisms following internal and external perturbations in either static or dynamic tasks with quantitative data [6]. It is commonly used to evaluate standing balance in stroke patients, but less commonly for sitting balance. In stroke patients, the posturographic parameters are usually obtained through force platforms to achieve the center of pressure (COP) trajectories. The analysis of COP parameters showed a larger sway area and displacements and less sample entropy for the stroke group than for the healthy controls [7]. Visual deprivation or sitting on an unstable surface is frequently used to increase the challenge and highlight the reduction of postural control [8]. The seated posturographic parameters also had a moderate correlation with other clinical balance function tests, such as the Trunk Impairment Scale (TIS) or the Berg Balance Scale (BBS) [8, 9].

The above study results support the clinical usefulness of sitting posturographic parameters in the balance assessment among stroke patients. Nonetheless, most of the previous studies used a force platform system to obtain seated posturography, but specific designs usually need to be adapted [8–10], unlike for the evaluation of standing balance. Therefore, they are mostly limited to research settings or fixed locations and are relatively costly. Instead, some researchers use an electromagnetic sensor or optoelectronic markers to record trunk sway or kinematics [11, 12], but the costs and technological complexity are also high. With the intent of obtaining a simple and feasible measurement for postural sway, we developed tracker-based posturography to measure trunk sway with a commercial virtual reality (VR) system (VIVE Pro, by HTC Inc., Taiwan) [13]. This system constitutes three VIVE trackers, which were originally used in VR gaming. The position and orientation signals of the trackers were documented to be in high agreement with a motion capture system (average translational error of 0.68 ± 0.32 cm) and a Polhemus Liberty magnetic tracking system (average translational error of 1.7 ± 0.4 mm [14, 15]. The trunk displacements (TDs) recorded through the lumbar tracker also have a moderate to high test–retest reliability and moderate to good correlation with the COP parameters during quiet standing [13]. This design has the potential to be applicable for sitting balance evaluation, but its reliability and validity have yet to be tested, especially among the potential target groups with impaired sitting balance. The purpose of this study was to test the reliability of tracker-based posturography for evaluating postural sway while sitting and to test the feasibility and validity in stroke patients. We added visual deprivation and unstable surfaces to differentiate the requirement of trunk postural control [8]. We hypothesized that the TD parameters in seated posture obtained by this system had fair to good test–retest reliability and at least moderate correlation with balance function tests. Moreover, the TD parameters would be able to discriminate among different sitting conditions and between the stroke patients and the controls. The results would help to extend the clinical application of the system in balance evaluation and rehabilitation.

## Methods

### Study design and participants

This was a cross-sectional study. Individuals with stroke were invited from in-patient wards or outpatient clinics in the Department of Physical Medicine and Rehabilitation of a university hospital in northern Taiwan. The inclusion criteria were as follows: aged 20 to 80 years old; diagnosed with stroke; medically and neurologically stable; and could sit unsupported for at least 10 min. The exclusion criteria were: unable to cooperate with the testing due to cognitive or speech disturbance. A convenient sample of healthy adults was recruited from the studying institute as the control group. The control group was used for reliability and discriminative validity testing, so we did not match age or gender given the difficulties associated with the recruitment of participants outside the hospital during the pandemics. They had no known history of visual, cognitive, cardiovascular, neurological or musculoskeletal problems and could walk normally. For the test–retest reliability test, we estimated that 22 participants were needed based on an intraclass correlation coefficient (ICC) = 0.5 and two observations per participant with power = 80% and α = 0.05 [16]. For comparison between the stroke and the controls, a minimum sample size of 42 participants (21 per group) should have 80% power to detect differences with α = 0.05 and an assumed effect size of 0.8 [17], according to G*Power 3.1.9.7 [18]. This study was approved by the Ethical Committee of the National Taiwan University Hospital (approval number: 202012050RIND, date of approval: 15/01/2021), and written informed consent was obtained prior to participation.

### Procedure of sitting balance test

The assessments of sitting stability were performed in a quiet laboratory in four sitting conditions, defined by the surface of the seat and the presence or absence of visual input: solid surface with eyes open (S-EO), solid surface and eyes closed (S-EC), soft and unstable surface with eyes open (U-EO), and soft and unstable surface with eyes closed (U-EC) [8]. For the solid surface condition, the participant sat on a stool with flat wooden seat that was 43 cm *42 cm in size and 41 cm high. For the soft and unstable surface condition, we mounted on the chair seat with a pre-inflated polyvinyl chloride disc cushion (Thera-Band model HYGE-00070, Akron, USA,) with a diameter of 33 cm and a height of 5 cm. No backrest was provided and the seated position was adjusted so that the participants sat with the knee flexed at 90 degrees and feet flat on the floors at hip width. The feet positions were marked to ensure a fixed position for each trial, and a visual target was provided 1.5 m ahead for the eyes-open conditions. The instruction to the participants was to cross their arms in front of the chest and keep upright and still as possible. A 60-s resting period was provided between each test. One researcher sat by the participants to ensure the safety during testing in case they tended to fall. The testing order of the four conditions was randomized and two tests of 60 s were conducted for each condition in one trial. The recording of the TD path was started manually and stopped automatically according to the pre-set testing duration. The first 22 participants were invited for the same-day retesting, repeating the above trial in the same order later the same day.

### VIVE tracker-based posturography

**Fig. 1:**
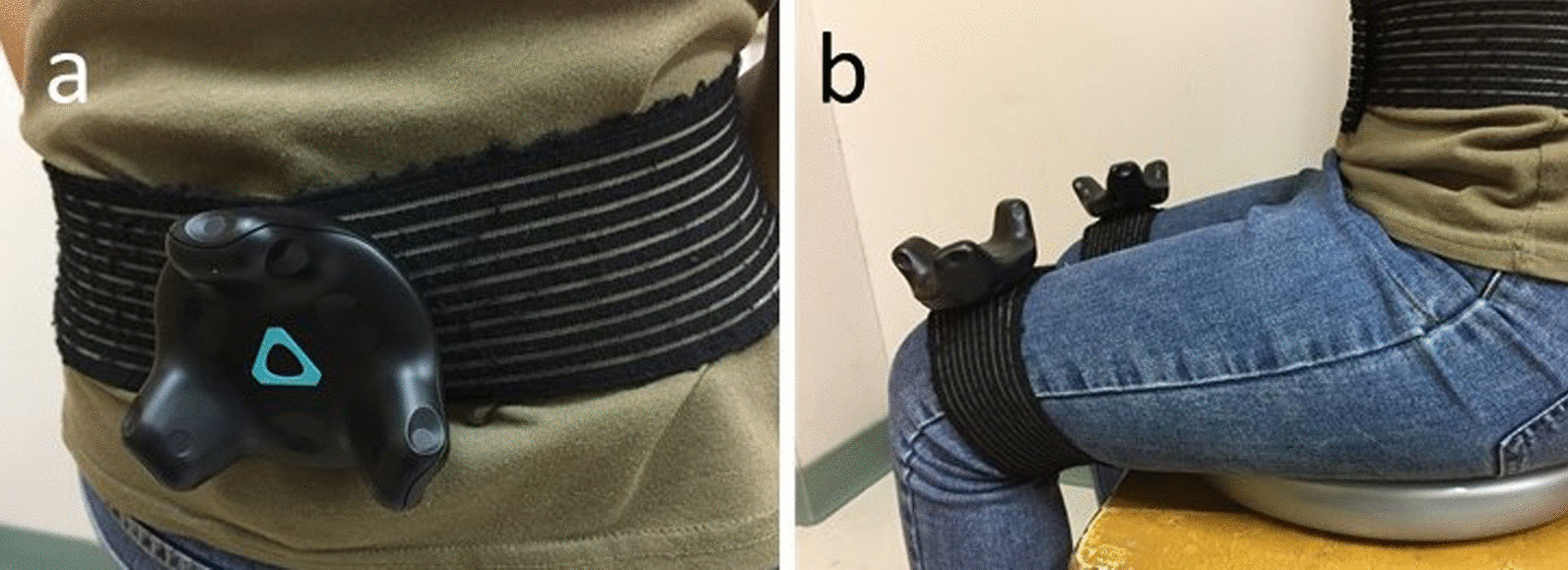
The setup of trackers for body position data collection. The trackers were docked with a standard tripod cradle head on elastic straps and positioned in the lumbar area at the L3 level **a** and on each distal thigh (**b**)

We used the VIVE Pro system (HTC Inc., Taiwan), which included two infrared laser emitter units (lighthouses, SteamVR Base Stations V2.0) and three wireless trackers (Steam VR Tracking V1.0). The head-mounted display was not worn in the current study. The details of the VIVE-tracker posturography setup have been described previously in a validation test in standing tasks [13]. In the current study, the VIVE tracker positions were modified with two trackers positioned on distal thigh above the patella and one in the lumbar area at the level around L3 (Fig. 1). The lumbar tracker data were used to represent the TD and the position was selected for the high reliability of pelvis displacement with a motion capture system during sitting tasks [19]. A custom C# script and the SteamVR (Valve Corp, Washington, USA) plugin for Unity3D was used to provide integration with HTC VIVE to record the position and orientation of the trackers with a sampling rate of 100 Hz. The displacements of the lumbar tracker in the horizontal plane were recorded as a bivariate (medio-lateral, ML, antero-posterior, AP) time series according to the local coordination system established with the aid of the two trackers at the distal thighs. The time series of the TD path was then used to compute the posturography parameters after passing through a fourth-order, zero phase Butterworth low-pass digital filter with a 5-Hz cutoff frequency in MATLAB (MathWorks Inc, Massachusetts, USA). Five TD parameters were computed with the equations used to calculate COP parameters [20], in which these parameters were categorized into different groups with low correlations between each other.

1. Root mean square (rms) distance in the AP direction (RDIST_AP_) and rms distance in the ML direction (RDIST_ML_): the standard deviation in the AP and ML time series from the mean TD;
2. Mean velocity in the AP direction (MVELO_AP_) and mean velocity in the ML direction (MVELO_ML_): the average velocity of the TD in the AP and ML directions;
3. 95% confidence ellipse area (AREA-CE): the area of the 95% bivariate confidence ellipse, which was expected to enclose approximately 95% of the points on the sway path.

### Balance function evaluation

The balance function of the stroke group was tested based on three performance-based clinical examinations: the BBS [21], Postural Assessment Scale for Stroke Patients (PASS)[22], and the Function in Sitting Test (FIST)[23] (Table 1). The former two tests were global balance function measures and the latter was a sitting-specific measure. The clinical tests were conducted by either author (HWL and TLT) according to standardized procedures. To mitigate the ceiling effect, one-leg standing and tandem standing in the BBS were performed with the affected leg as the weight-bearing leg [24].

**Table 1:** The profiles of three performance-based balance tests used in the current study

Profiles	BBS	PASS	FIST
Total item number	14	12	14
Number of items assessing static sitting balance	1	1	6
Number of items assessing dynamic tasks in sitting	0	0	8
Number of items assessing postural change related to sitting	3	3	0
Scale	5-point	4-point	5-point
Scoring	0–56	0–36	0–56

*BBS* Berg Balance Scale, *PASS* Postural Assessment Scale for Stroke Patients, *FIST* Function in Sitting Test

### Statistical analysis

The descriptive statistics of five TD parameters were calculated from the average of two trials and normality of the data distribution was assessed using the Shapiro–Wilk test. The same day test–retest reliability was tested with the ICC from the average data of 2 tests in each trial. The ICC estimates and their 95% confident intervals were based on a single rating, absolute-agreement, 2-way mixed-effects model for each condition. An ICC higher than 0.75 was considered excellent, between 0.6 and 0.74 was good, between 0.4 and 0.59 was fair and less than 0.4 was poor [25]. The difference in the TD parameters between the two groups of participants and among four conditions within each group was compared with the Mann–Whitney U test and the Friedman test if a normal distribution was violated. The *p* values for the post-hoc examination among multiple comparisons of tasks were examined with the Wilcoxon signed rank test and adjusted using the Bonferroni correction. In addition, a generalized estimating equation (GEE) was used to examine the influence of the group and task on the changes in the TD parameter. We assumed an exchangeable correlation structure and included the main effects of group and task as well as the interaction between them. Finally, we examined the correlation between the sway parameters and the clinical balance function tests with Spearman’s ρ or Pearson’s correlation coefficient, depending on the distribution of the data. The size of the correlation coefficient was interpreted as being very high (0.90), high (0.7 to 0.9), moderate (0.5 to 0.7) or low (0.3 to 0.5)[26]. Statistical analyses were performed using SPSS 15.0 for Windows (SPSS Inc., Chicago, USA) with a statistical significance of p < 0.05.

## Results

We recruited 21 stroke patients (76.2% male) and 21 controls (47.6% male). Their age, body height and weight were 58.6 ± 10.0 years, 166.0 ± 6.3 cm, and 69.0 ± 12.3 kg for the stroke group, and 32.2 ± 8.0 years old, 169.4 ± 8.1 cm, and 64.0 ± 14.1 kg for the controls. Twenty-two participants had a retest and their average age was 34.1 ± 8.9 years old, with 40.9% of them being female and 18.2% being stroke patients. Approximately two-thirds of the stroke group had ischemic stroke, and the median disease duration was 25.9 months (Table 2). Two thirds of them were independent ambulators and the average scores of the BBS, the PASS and the FIST were 70%, 84% and 97% of the best scores respectively. The proportions achieving a ceiling score were 4.8%, 4.8% and 57.1% for the BBS, PASS and FIST, respectively. All of the participants completed the tests, except for one stroke subject with a BBS of 10 who failed the 1-min quiet sitting on a soft surface test.

**Table 2:** Basic data and balance function of the stroke group

Variable	Result
Ischemic stroke (n, %)	14 (66.7%)
Hemiplegic side, right (n, %)	12 (57.1%)
Onset time, months (median, interquartile range)	25.9,110.6
Ambulation status (n, %)	
Dependent on physical assistance	3 (14.3%)
Dependent on supervision	3 (14.3%)
Independent level surface only	1 (4.8%)
Independent	14 (66.7%)
Berg balance scale (median, interquartile range)	43, 20
Postural assessment for stroke scale (median, interquartile range)	31, 5
Function in Sitting test (median, interquartile range)	56, 4

**Fig. 2:**
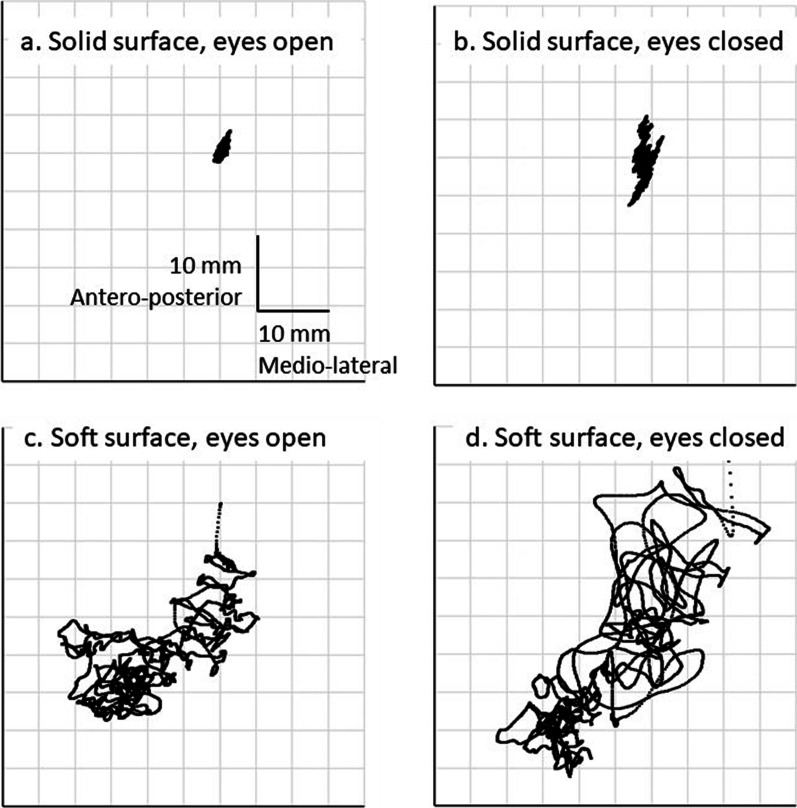
An example of the antero-posterior and medio-lateral displacement path of the trunk in a stroke patient with left side weakness during the four sitting conditions

Fig. 2 shows an example of the TD trajectories of one stroke participant in four sitting conditions. All the TD parameters violated the assumption of a normal distribution; therefore, nonparametric methods were used for all the analyses. The repeatability was fair to excellent in most TD parameters according to ICCs (Table 3), except for the S-EO condition. AREA-CE had excellent reliability in all the conditions, except for the U-EC condition. In the S-EC and U-EO conditions, ICCs ranged from 0.51 to 0.90 for all TD parameters, and the ML directions had generally higher reliability than the AP directions for the distance and velocity parameters. Poor reliability (ICCs lower than 0.4) was observed for RDIST_ML_ and MVELO_ML_ in S-EC, and for AREA-CE in U-EC.

**Table 3:** Intraclass correlation coefficients (95% confidence intervals) for trunk displacement parameters across four conditions

	Solid surface		Soft surface	
	Eyes open	Eyes closed	Eyes open	Eyes closed
RDIST_AP_ (mm)	0.81*** (0.58, 0.92)	0.74*** (0.47, 0.88)	0.51** (0.13, 0.76)	0.60** (0.26, 0.81)
RDIST_ML_ (mm)	0.28 (− 0.11, 0.61)	0.79*** (0.57, 0.91)	0.80*** (0.58, 0.91)	0.64** (0.31, 0.83)
MVELO_AP_ (mm/s)	0.52** (0.13, 0.77)	0.54** (0.15, 0.78)	0.64*** (0.27, 0.84)	0.76*** (0.51, 0.89)
MVELO_ML_ (mm/s)	0.12 (− 0.20, 0.47)	0.72*** (0.45, 0.88)	0.75*** (0.45, 0.89)	0.53** (0.14, 0.77)
AREA-CE (mm^2^)	0.76 *** (0.45, 0.90)	0.90*** (0.79, 0.96)	0.89*** (0.76, 0.95)	0.33 (− 0.11, 0.66)

*RDIST*_*ML*_ Root mean square distance in the medial–lateral direction, *RDIST*_*AP*_ Root mean square distance in the anterior–posterior direction, *MVELO*_*ML*_ mean velocity in the medio-lateral direction, MVELO_AP_ in the antero-posterior direction, *AREA-CE* 95% confidence ellipse area

Significance level of the ICC: *p < 0.05; **p < 0.01; ***p < 0.001;

There was a trend of incremental increase in the TD parameters, with the order of S-EO, S-EC, U-EO and U-EC (Table 4). The Friedman test showed a mostly significant difference among the four sitting conditions within each group for most TD parameters, with the only exceptions of MVELO_AP_ among the control group. In addition, a significant difference was observed only between sitting on a solid surface and sitting on a soft surface, but not between the eyes open and eyes closed conditions while sitting on the same surface according to post-hoc analysis with the Wilcoxon signed rank test and the Bonferroni correction (p < 0.0083). AREA-CE and RDIST_ML_ could differentiate this difference effectively. In addition, the incremental difference in TD parameters within each group had a trend to be more apparent for stroke group, with the exception of MVELO_AP_.

**Table 4:** The median (interquartile range) of all trunk displacement parameters and the results of comparison in the two groups

Sway parameters	Groups	Solid、eyes open	Solid、eyes closed	Soft、eyes open	Soft、eyes closed	*p value
RDIST_AP_ (mm)	Stroke	1.69 (1.51)^a^	1.73 (1.17)^b^	2.32 (2.09)	3.18 (3.18)^a,b^	0.001
	Control	0.94 (0.31)	0.86 (0.68)	1.16 (1.03)	1.17 (0.78)	0.036
	^&^p value	0.001	0.001	0.001	< 0.001	
RDIST_ML_ (mm)	Stroke	0.62 (0.39)^a^	0.71 (0.49)^b^	2.12 (1.80)^a,b^	2.25 (0.96)^a,b^	< 0.001
	Control	0.41 (0.17)^a^	0.45 (0.33)^b^	1.21 (0.74)^a,b^	1.19 (0.67)^a,b^	< 0.001
	^&^p value	0.002	0.056	0.009	< 0.001	
MVELO_AP_ (mm/s)	Stroke	3.38 (0.66)	3.46 (0.92)	4.07 (1.24)	4.23 (1.80)	< 0.001
	Control	3.21 (0.48) ^a^	3.38 (0.80)	3.71 (0.85) ^a^	3.60 (0.73) ^a^	0.072
	^&^p value	0.218	0.782	0.151	0.029	
MVELO_ML_ (mm/s)	Stroke	1.99 (0.85)^a^	2.01 (0.79)^b^	3.09 (1.18)^a^	3.30 (1.35)^a,b^	< 0.001
	Control	1.88 (0.15)^a^	1.84 (0.47)	2.12 (0.57)	2.20 (0.48)^a^	0.013
	^&^p value	0.345	0.195	< 0.001	< 0.001	
AREA-CE (mm^2^)	Stroke	14.45 (18.15)^a^	20.01 (17.65)^b^	81.78 (126.14)^a,b^	89.44 (124.95)^a,b^	< 0.001
	Control	4.35 (2.37)^a^	5.78 (7.49)^b^	24.64 (19.86)^a,b^	23.70 (20.12)^a,b^	< 0.001
	^&^p value	< 0.001	< 0.001	0.001	< 0.001	

*p value: Comparison within the sitting tasks according to the Friedman test; ^&^p value: Comparison between the groups according to Mann–Whitney U test

^a,b^Paired comparison with significant difference according to Wilcoxon signed rank test and a Bonferroni correction

Between-group comparisons between the stroke and control groups showed significant differences in all TD parameters in U-EO and U-EC, but the results were less consistent in S-EC and S-EO according to the Mann–Whitney U test. RDIST_AP_ and AREA-CE were the only two TD parameters to differentiate between the two groups in all sitting conditions. Meanwhile, MVELO_AP_ could discriminate between the two groups only in U-EC. Further analysis of the effects of group and task was conducted with a GEE analysis (Table 5). There was a significant main effect of sitting conditions on all TD parameters, with significant increases in U-EC and U-EO. A main effect of group was significant for RDIST_AP_ (Wald χ^2^ = 13.76, p < 0.001), RDIST_ML_ (Wald χ^2^ = 6.28, p = 0.01) and AREA-CE (Wald χ^2^ = 24.91, p < 0.001), while it was nonsignificant for MVELO_AP_ and MVELO_ML_. Nonetheless, the interaction effect of group and tasks was significant for these parameters, indicating that the group effect was significant for the velocity parameters only in the unstable sitting condition but not in the stable sitting condition.

**Table 5:** The effect of group and task on the trunk displacement parameters according to the generalized estimating equation (GEE) analysis

Parameter	β	Standard error	Wald χ^2^	95% confidence limits	p
RDIST_AP_					
Intercept	0.98	0.08	159.24	0.83–1.13	< 0.001
Group	0.93	0.25	13.76	0.44–1.42	< 0.001
Task (*vs* solid, eyes open)					
Solid, eyes closed	0.09	0.12	0.55	− 0.15–0.32	0.46
Soft, eyes open	0.43	0.17	6.23	0.09–0.77	0.01
Soft, eyes closed	0.27	0.10	6.87	0.07–0.47	0.01
Group*Task (*vs* control*eyes open)					
Stroke* soft, eyes closed	1.29	0.45	8.09	0.40–2.17	< 0.01
Stroke* soft, eyes open	0.48	0.37	1.67	− 0.25–1.20	0.20
Stroke* solid, eyes closed	− 0.10	0.24	0.16	− 0.57–0.37	0.69
RDIST_ML_					
Intercept	0.46	0.04	121.65	0.38–0.54	< 0.001
Group	0.28	0.11	6.28	0.06–0.50	0.01
Task (*vs* solid, eyes open)					
Solid, eyes closed	0.14	0.06	5.37	0.02–0.25	0.02
Soft, eyes open	0.92	0.15	36.70	0.63–1.22	< 0.001
Soft, eyes closed	0.82	0.11	54.95	0.60–1.04	< 0.001
Group*Task (*vs* control*eyes open)					
Stroke* solid, eyes closed	0.15	0.33	0.20	− 0.5–0.79	0.66
Stroke* soft, eyes open	0.60	0.31	3.68	− 0.01–1.21	0.06
Stroke* soft, eyes closed	0.94	0.31	9.19	0.33–1.55	< 0.01
MVELO_AP_					
Intercept	3.12	0.15	431.20	2.83–3.42	< 0.001
Group	0.56	0.41	1.89	− 0.24–1.37	0.17
Task (*vs* solid, eyes open)					
Solid, eyes closed	0.51	0.30	2.87	− 0.08–1.10	0.09
Soft, eyes open	0.37	0.11	12.27	0.16–0.58	< 0.001
Soft, eyes closed	0.46	0.13	12.95	0.21–0.71	< 0.001
Group*Task (*vs* control*eyes open)					
Stroke* solid, eyes closed	− 0.62	0.50	1.55	− 1.60–0.36	0.21
Stroke* soft, eyes open	− 0.07	0.35	0.04	− 0.77–0.62	0.83
Stroke* soft, eyes closed	0.24	0.39	0.40	− 0.51–1.00	0.53
MVELO_ML_					
Intercept	1.87	0.04	2077.44	1.79–1.95	< 0.001
Group	0.33	0.20	2.71	− 0.06–0.72	0.10
Task (*vs* solid, eyes open)					
Solid, eyes closed	0.19	0.16	1.39	− 0.13–0.50	0.24
Soft, eyes open	0.27	0.08	10.03	0.10–0.43	< 0.01
Soft, eyes closed	0.42	0.12	12.81	0.19–0.65	< 0.001
Group*Task (*vs* control*eyes open)					
Stroke* solid, eyes closed	0.16	0.54	0.09	− 0.89–1.22	0.76
Stroke* soft, eyes open	0.82	0.30	7.45	0.23–1.41	< 0.01
Stroke* soft, eyes closed	1.08	0.37	8.38	0.35–1.82	< 0.01
AREA-CE					
Intercept	5.42	0.67	66.22	4.12–6.73	< 0.001
Group	12.99	2.60	24.91	7.89–18.09	< 0.001
Task (*vs* solid, eyes open)					
Solid, eyes closed	1.23	1.10	1.26	− 0.92–3.38	0.26
Soft, eyes open	23.84	4.96	23.11	14.12–33.56	< 0.001
Soft, eyes closed	19.68	3.56	30.58	12.70–26.65	< 0.001
Group*Task (*vs* control*eyes open)					
Stroke* solid, eyes closed	17.83	17.12	1.08	− 15.73–51.38	0.30
Stroke* soft, eyes open	63.33	20.30	9.74	23.55–103.11	< 0.01
Stroke* soft, eyes closed	95.21	26.01	13.40	44.23–146.20	< 0.001

*RDIST*_*ML*_ Root mean square distance in the medial–lateral direction, *RDIST*_*AP*_ Root mean square distance in the antero-posterior direction, *MVELO*_*ML*_ mean velocity in the medio-lateral direction, MVELO_AP_ in the anterior–posterior direction, *AREA-CE* 95% confidence ellipse area

Spearman's *ρ* coefficients were computed between each postural parameter and each balance performance scores for the stroke group (Table 6). The PASS had a moderate to high correlation with MVELO_AP_ and MVELO_ML_ in both S-EO and S-EC (Spearman's *ρ* = -0.49 to -0.73) and mostly low to moderate in U-EO and U-EC (Spearman's *ρ* = -0.21–0.51). Meanwhile, the BBS was correlated with these two TD parameters in S-EO and U-EO (Spearman's *ρ* = -0.38 to -0.75). For FIST, we observed only a moderate correlation with MVELO_AP_ (Spearman's *ρ* = -0.58) in U-EO. The other TD parameters, including RDIST_AP_, RDIST_ML_, and AREA-CE, were not correlated with these balance performance scales.

**Table 6:** Spearman’s *ρ* values between the trunk displacement parameters from the VIVE trackers and the results of balance performance tests

	Solid surface			Soft surface		
	BBS	PASS	FIST	BBS	PASS	FIST
Eyes open						
RDIST_AP_	− 0.15	− 0.15	− 0.18	− 0.20	− 0.28	− 0.11
RDIST_ML_	− 0.28	− 0.35	0.24	− 0.20	− 0.19	− 0.24
MVELO_AP_	− 0.68***	− 0.73***	− 0.27	− 0.56*	− 0.51*	− 0.58**
MVELO_ML_	− 0.75***	− 0.73***	− 0.41	− 0.38	− 0.29	− 0.50*
AREA-CE	− 0.28	− 0.27	− 0.00	− 0.13	− 0.14	− 0.21
Eyes closed						
RDIST_AP_	− 0.12	− 0.16	0.03	0.10	− 0.08	0.12
RDIST_ML_	− 0.06	− 0.17	0.41	− 0.09	− 0.13	− 0.15
MVELO_AP_	− 0.36	− 0.49^*^	0.09	− 0.34	− 0.44	− 0.17
MVELO_ML_	− 0.41	− 0.53^*^	− 0.03	− 0.17	− 0.21	− 0.17
AREA-CE	− 0.13	− 0.16	0.25	0.09	− 0.04	0.12

*RDIST*_*ML*_ Root mean square distance in the medial–lateral direction, *RDIST*_*AP*_ Root mean square distance in the antero-posterior direction, *MVELO*_*ML*_ mean velocity in the medio-lateral direction, MVELO_AP_ in the anterior–posterior direction, *AREA-CE* 95% confidence ellipse area

*p < 0.05; **p < 0.01; ****p < 0.05

## Discussion

We evaluated the reliability and validity of a VIVE tracker-based posturography for evaluating sitting balance among stroke patients and the normal controls. Our results confirmed that the TD parameters had a mostly fair to good test–retest reliability, except for distance and velocity measures in S-EO. These parameters could discriminate between the stroke patients and healthy controls, and between those who sat on stable or unstable surfaces. Meanwhile, MVELO_AP_ and MVELO_ML_ had moderate to strong correlations with the BBS and the PASS while sitting on solid surfaces among stroke individuals. Therefore, using this setup for postural evaluation in seated posture was feasible. Since this was a novel design for sitting posture evaluation, we compared our results with the previous studies obtaining posturography with other methods, mainly force platform systems.

The reliability of this setup was tested among young healthy adults, with an ICC between 0.56 and 0.90 across four standing tasks and the highest in MVELO_ML_ [13]. In the current study, we demonstrated generally fair to good reliability for most of the sitting tasks, and the ICCs were mostly above 0.5, except for a few parameters in the S-EO condition. The results were comparable to previous studies that used force platforms to obtain a seated stabilogram among healthy adults or individuals with stroke, low back pain and spinal cord injuries [9, 19, 27–30]. Most studies showed that mean velocity and in the ML direction had the highest ICC among many postural parameters. In contrast, we observed poor reliability for MVELO_ML_ and RDIST_ML_ in the S-EO condition, which was the most stable condition and was not tested in most of the above studies. There were several possible reasons for low ICCs, including the short duration of the measurements, the small sample size, measurement errors and lack of variability [31, 32]. However, we hypothesized that the specifically low ICC under the S-EO condition was attributed to the lack of variability under this condition as observed in other studies [9, 27]. Comparatively, the body sway size and velocity were smaller during quiet sitting than standing [33], which was supported by comparing our current results in sitting and previous study results in standing with the same setup [13]. Moreover, the stability was higher in the ML direction than in the AP directions, rendering a low between-subject variance and low ICC for RDIST_ML_ and MVELO_ML_ in S-EO, as evidenced by the small interquartile range. This issue may be less significant in subjects with lower trunk stability or the sitting conditions with higher challenges, such as sitting on unstable surfaces as in most of the previous studies. However, it is noteworthy that the participants with inadequate trunk balance may have difficulty completing seated balance tests on unstable surfaces. Therefore, increasing repetition should be considered to ensure a higher reproducibility in the S-EO condition if low between-subject variability is expected [19].

We tested four sitting conditions with the combination of changes in vision and sitting surfaces, which are commonly used to challenge the trunk control ability during quasi-static posture. Friedman tests showed mostly significant differences for all TD parameters, except for MVELO_AP_ for the control group. Despite the incremental increase of the TD parameters from S-EO, S-EC, U-EO and U-EC, the pairwise comparisons between each sitting task were less apparent among the control group. In addition, the effect of eye closure was generally nonsignificant between sitting on the same sitting surface in both groups. Although visual input is an important contribution stabilize posture, its role in less challenging tasks or in young populations is less important [10, 20]. In addition, our design with the participants seated with both feet on ground also provided a stabilizing effect in the AP direction and likely diminished the destabilizing effect of eye closure [34]. In contrast, the effect of unstable surfaces on sitting sway was universally significant for all TD parameters, and was not limited to the ML direction [8, 35]. It is likely to be related to the unstable surface we used, a soft cusion that gives the sitting plane with three-degrees of freedom instead of one [35].

We tested the discriminative validity of the measurement system to differentiate between two groups with a known difference in balance. We were able to discriminate two groups with all parameters under U-EO and U-EC conditions and with parts of the parameters under S-EO and S-EC conditions. In addition, we were aware that the difference between the two groups could be attributed to the effect of both stroke and age. The effect of age on trunk control while sitting has rarely been explored, except for during seated dynamic tasks [35]. Our stroke participants exhibited relatively good sitting balance given that more than half of them achieved a ceiling score with FIST. Among these TD parameters, stroke group had a main effect on the RDIST_AP_, RDIST_ML_ and AREA-CE and an interaction effect with sitting tasks on RDIST_AP_, RDIST_ML_ MVELO_ML_ and AREA-CE. The interaction effect was significant for sitting on soft surface. These findings were generally consistent with previous studies, i.e., stroke patients had increased seated postural instability compared with normal controls with an interaction effect from vision and sitting surfaces [7, 8], with greater impact in the ML direction than in the AP direction [8]. Moreover, we postulated that the impact from stroke was greater than that from age, according to a previous study comparing the standing balance in three groups of young healthy, old healthy and stroke participants [36]. These researchers observed an effect of stroke on increasing postural instability in the ML direction and an effect of age on an increase in the AP direction during standing, which represents a different, but supposedly more challenging task than sitting. Future studies with three groups of subjects should be performed to clarify the effects of age and stroke on sitting balance.

The TD parameters were also validated against three clinical balance measures, namely the BBS, the PASS and the FIST. Alterations in the postural control system can be reflected in changes in COP parameters, but there are only a limited number of studies exploring the correlation between clinical and instrumental balance assessments for sitting or standing stability in stroke patients [8, 9, 37]. Most of the studies selected overall balance performance, not a sitting-specific measure for comparison. For example, high Spearman's correlations (0.72, 0.79) were found between the TIS and the frontal and sagittal excursions during sitting, and low correlations were found between the TIS and the sway area and sway velocity [9]. Meanwhile, lateral balance control showed the strongest association with the BBS [8]. According to our results, both MVELO_AP_ and MVELO_ML_ had exhibited moderate to high correlations with the balance function tests, and the correlation coefficient was the highest with the PASS, followed by the BBS and the FIST. There was also a trend of higher correlation with the BBS and PASS while sitting on solid surface. In contrast, FIST was significantly correlated with MVELO_AP_ and MVELO_ML_ only during the U-EO condition, probably because of the high ceiling effect. Since an increase in COP velocity represents a decreased ability to control posture [38], it may explain the predictive ability of sitting balance for ambulation or functional outcomes, which is usually highly correlated with the overall balance performance tests [4]. While both functional tests and instrumented measures can monitor balance function in stroke subjects, the former is the main tool in clinical settings. Nonetheless, the precise balance characteristics described by clinical balance evaluations are not easy to define, and their items for assessing sitting balance are less appraised [5]. Sitting posturographic parameters help provide quantitative assessment of poststroke balance and target on training and predict independent walking abilities even when stroke patients cannot reach their arms forward or stand upright [8, 39]. They also had no ceiling effect, which was documented in the current study using the FIST and a previous study using the BBS among stroke patients undergoing inpatient rehabilitation [40]. Some authors suggest combining quantitative posturography and clinical evaluation whenever possible to achieve a comprehensive postural impairments and disabilities in stroke patients [37]. Our setup may have advantages over platform systems in terms of availability, portability and cost.

## Limitations

We would like to address two limitations of this study. First, although we have documented the discriminative validity of the system between two groups with or without known balance problem, the discriminative ability to differentiate stroke patients and age-matched healthy control group has not been confirmed. Second, one of the inclusion criteria for the stroke group was the ability to sit unsupported for at least 10 min, which limits the generalization of the results to patients with worse balance. Adjustment of the test duration and repetition may be required to ensure reliability, but we believe the measurement should still be applicable.

## Conclusion

In conclusion, this tracker-based posturography method provides a feasible means of measuring stroke patients’ seated stability with acceptable reliability and validity. The results were generally comparable to the data using kinetic data of the COP, although the postural sway recorded by the setup represented different postural behaviors. This posturography used commercially available hardware and could provide evaluation for both quasi-static sitting and standing tasks. It also has the potential to integrate the visual environment to create challenges or perturbations for balance evaluation or training [41]. Further studies in various populations should be conducted to evaluate the responsiveness of the parameters to characterize the progress for stroke patients during rehabilitation.

## Data Availability

The datasets used and/or analyzed during the current study are available from the corresponding author on reasonable request.

## Abbreviations

AP: Antero-posterior
AREA-CE: 95% Confidence ellipse area
BBS: Berg balance scale
COP: Center of pressure
U-EC: Soft surface and eyes closed
U-EO: Soft surface and eyes open
FIST: Function in sitting test
GEE: Generalized estimating equation
ICC: Intraclass correlation coefficient
ML: Medio-lateral
MVELO_AP_: In the anterior–posterior direction
MVELO_ML_: Mean velocity in the medial–lateral direction
PASS: Postural Assessment Scale for Stroke Patients.
RDIST_AP_: Root mean square distance in the anterior–posterior direction
RDIST_ML_: Root mean square distance in the medial–lateral direction
S-EC: Solid surface and eyes closed
S-EO: Solid surface and eyes open
TD: Trunk displacement
TIS: Trunk Impairment Scale
VR: Virtual reality
